# The Contribution of the Dorsolateral Prefrontal Cortex in Full and Divided Encoding: A Paired-Pulse Transcranial Magnetic Stimulation Study

**DOI:** 10.3233/BEN-2010-0273

**Published:** 2010-11-23

**Authors:** Sophie Blanchet, Geneviève Gagnon, Cyril Schneider

**Affiliations:** ^1^Center for Interdisplinary Research in Rehabilitation and Social IntegrationQuebec CityCanada; ^2^School of PsychologyUniversité LavalQuebec CityQCCanada; ^3^CHUQ Research CenterDepartment of RehabilitationUniversité LavalQuebec CityQCCanada

**Keywords:** Episodic memory, attentional resources, verbal material, visuospatial material, hemispheric asymmetry, transcranial magnetic stimulation (TMS)

## Abstract

This research investigated the contribution of the dorsolateral prefrontal cortex (DLPFC) in the attentional resources in episodic encoding for both verbal and non-verbal material. Paired-pulse transcranial magnetic stimulations (TMS) were used to interfere transiently with either the left or right DLPFC during encoding under full attention (FA) or under divided attention (DA) in a recognition paradigm using words and random shapes. Participants recognized fewer items after TMS over the left DLPFC than over the right DLPFC during FA encoding. However, TMS over the left DLPFC did not impair performance when compared to sham condition. Conversely, participants produced fewer items after TMS over the right DLPFC in DA encoding compared to sham condition, but not compared to TMS over the left DLPFC. These effects were found for both words and random shapes. These results suggest that the right DLPFC play an important role in successful encoding with a concomitant task regardless of the type of material.

## 1. Introduction

Episodic memory allows an individual to remember the temporal and spatial context of events [29]. Successful encoding into episodic memory depends on the availability of attentional resources. Accordingly, divided attention (DA) during encoding has a detrimental effect on subsequent recall performance in healthy young individuals [2,14,22]. The decreased performance following encoding under DA compared to encoding under full attention (FA) would reflect limitations of control processes that depend on the prefrontal (PFC) areas. Left PFC is often activated in episodic encoding under FA regardless of the type of material (see [12] for a review). The dorsolateral PFC (DLPFC) subareas have been reported to be sensitive to the DA detrimental effect during encoding. Indeed, using positron emission tomography (PET), decreased left DLPFC activations have been found during encoding when semantic organizational or associative strategies were explicitly required in encoding concomitantly to a secondary task [17,22]. Only verbal material was used in the previous DA studies. Non-verbal material that is novel without any pre-existing representation, however, may solicit greater attentional processes than verbal material [6]. In addition, the previous findings rely on neuroimaging data, which use hemodynamic and metabolic indices. This may be an issue because

ISSN 0953-4180/10/$27.50 © 2010 – IOS Press and the authors. All rights reserved the activations do not mean that the activated structures are functionally necessary to perform the targeted cognitive task [42].

By inducing a safe and transient interference with transcranial magnetic stimulations (TMS) in healthy individuals, the mandatory role of a cortical area in a given cognitive task can be investigated. Memory performance disruption by TMS demonstrates thus that the stimulated area is critical to perform the task. Impaired recall performance was also observed following repetitive TMS (rTMS) over the left DLPFC during FA encoding of a story [35], semantically unrelated word pairs [20,41] and complex indoor/outdoor pictures [36]. Recall deficits, however, have been found after TMS over the right DLPFC during encoding of word pairs with high imagery content [41]; this may be indicative of the role that this region plays in image-based processing [41]. These previous TMS studies investigated the DLPFC role in episodic memory uniquely under FA but not in DA. Whether the DLPFC is required or not for successful encoding in DA remains unclear. Indeed, patients with anterior injuries did not suffer from a large detrimental effect of DA compared to those with posterior injury [48] or healthy controls [3]. However, in these studies, patients were pooled regardless of the injured frontal subareas that are known to play different functions. In addition, it cannot be ruled out that their cognitive profiles reflect the involvement of neural and/or cognitive compensatory processes occurring after injury.

We investigated the contribution of the left and right DLPFC in encoding according to the availability of attentional resources for both verbal and non-verbal material. To this end, paired-pulse TMS was applied to interfere transiently with the left or right DLPFC during encoding under FA or DA of random shapes or words. In reference to TMS or neuroimaging studies in encoding under FA [17,20,22,35,37], we expected that TMS over left DLPFC exert a detrimental effect on memory performance compared to TMS delivered over right DLPFC. Since previous neuroimaging studies found that a secondary task decreased the left frontal activity during encoding [2,22], the interferential effect of TMS on memory performance when applied over the left DLPFC should be less pronounced for encoding under DA.

## 2. Methods

### *2.1. Participants*

Sixteen healthy young participants (mean age, 22.75 $\pm$ 3.33; range, 18–30 years) with an average of 16.37 (2.89) years of formal education gave their written consent before taking part in the protocol. Participants were all right-handed. The mean of their laterality index is estimated at 83 (17.09) on the basis of Edinburgh Handedness Inventory [34]. The other inclusion criteria were normal or corrected-to-normal vision and French as the native language. The exclusion criteria were a history of psychiatric or neurological disorders (e.g., stroke, head injury) and alcoholism. None received anxiolytic or antidepressant treatments known to interfere with attention or memory. In respect of safety guidelines for the use of TMS [37,49], participants did not present any history of epilepsy, migraine, cardiovascular diseases, or metal in head or jaw. In compliance with the Declaration of Helsinki, the project was approved by the local Ethics Committees.

### *2.2. TMS memory paradigm*

The paradigm is illustrated in Figure 1. Participants were seated at a distance of 23 inches from a computer monitor. All stimuli were displayed in white on a black screen and each one was unique. The non-verbal material consisted of complex random shapes designed to be neither figurative nor verbalizable (see [7], for more details). The verbal material consisted of unrelated nouns of four- to eight-letters [15]. All lists of words were equivalent in frequency (4.46 $\pm$ 0.79) and imageability (5.19 $\pm$ 0.89). For each condition, one list of 20 targets was presented. For both verbal and non-verbal materials, there were six TMS conditions: (1) the right DLPFC was stimulated during FA encoding; (2) the left DLPFC was stimulated during FA encoding; (3) the right DLPFC was stimulated during DA encoding; (4) the left DLPFC was stimulated during DA encoding; (5) a sham TMS encoding condition under FA; (6) a sham TMS encoding condition under DA. During the encoding phase, each stimulus appeared for 1000 ms in the center of the screen, with a 2500 ms inter-stimulus interval. The encoding phase was separated from the recognition phase by a gray screen lasting 5000 ms. In the recognition phase, probes corresponding to half of the previously displayed items mixed with the same number of novel items appeared successively in the center of the screen. Each probe was presented for 1000 ms. The elapsed time between two successive probes was 3500 ms. Participants pressed the green key with the right index finger for a previously seen item and the red key with the left index finger for an item not seen previously. The position of the targets in the recognition phase was pseudo-random to avoid serial effects.

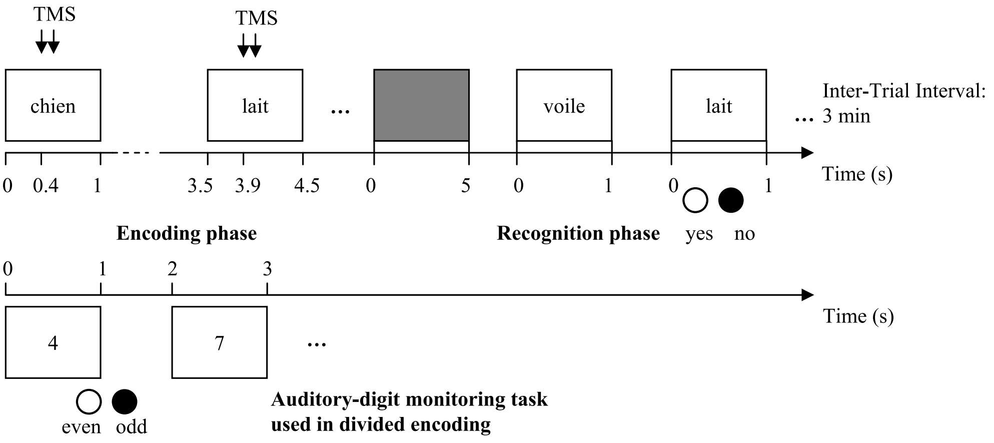

Fig. 1. Transcranial magnetic stimulations were applied during encoding under full or divided attention either on the left or the right dorsolateral prefrontal cortex.

Each list was separated by a pause lasting about 3 min. During the FA encoding phase, participants only memorized the stimuli (i.e., without any concurrent task). During the DA encoding phase, participants were instructed to divide their attention equally between the encoding task and a secondary task. The latter consisted of an auditory digit-monitoring task. Participants listened to series of odd and even digits at a rate of 1 digit every 2000 ms. They pressed the green key for an odd preceded by an even, the red key in other case. Key order was counterbalanced between participants. For each material, participants were trained on two lists of 8 items prior to the experimental conditions. There was one session for non-verbal material and one session for verbal material, each of which was administered on different days. Each session lasted approximately 90 minutes. Memory paradigms were run on E-Prime software in interface with the TMS system.

### 2.3. *TMS protocol*

Transcranial magnetic stimulations were applied to the scalp using a custom made 70-mm (wing diameter) double-cone coil connected to 2 Magstim 200 electromagnetic stimulators coupled with a Bistim module (Magstim Company Limited, Whitland, UK). The coil was positioned over the left or the right DLPFC corresponding respectively to the F3 and F4 scalp sites according to a reference grid (10–20 EEG system; [23]). F3 and F4 sites correspond to Broadmann's area 9 [45]. Double TMS pulses were induced at a fixed interstimulus interval (ISI). It is well documented that 3 to 5-ms ISIs induce an intracortical inhibition in the primary motor cortex [26] and interfere with higher cognitive functions (see [38] for a review). Therefore, we used 2 sub-threshold TMS elicited 3 ms apart to induce a transient interference in DLPFC processing. As previously applied in TMS memory studies [35,36, 41], TMS intensity was set at 90% motor-threshold. Motor-threshold was determined as the minimal intensity of the stimulator output enabling us to induce a motor evoked potential in the resting first dorsal interosseus higher than 50 $\mu$V for 5 out of 10 TMS trials [39]. Motor-thresholds were estimated for right and left motor cortices (means, 25.28 $\pm$ 2.42 and 25 $\pm$ 2.39, respectively). Each paired-pulse TMS was delivered 500 ms after each stimulus onset. We chose to induce paired-pulse TMS 500 ms after stimulus onset in encoding because DLPFC-related strategy processes occurred during this time-interval (e.g. [4,8]). For the sham encoding conditions under FA or DA, the TMS coil was placed above the interhemispheric scissure (at Cz) and was rotated 90 degrees tangentially to the head. Thus the participants kept hearing the discharge TMS noise, while elicited TMS did not penetrate into the scalp. These sham encoding conditions enabled us to control for the impact of the auditory noise (still audible at the same TMS intensity between all conditions). Unfortunately we cannot control the impact of the superficial epidermical reactions over the muscles of the scalp induced in the experimental TMS condition. The muscular contractions are, however, quite minimal when TMS are delivered under the subthreshold motor level as in our study.

### 2.4. *Data analysis*

Different response indices were analyzed to assess memory performance and strategies. In reference to the two-high threshold model [44], we analyzed hits (H), false alarms (FA) and discrimination rate (H–FA). The discrimination rate, that ensures that the participants discriminated correctly between the targets and the distractors during recognition, had to be greater than chance levels (Ø). The response time of hits was also considered. For each type of material, an ANOVA with repeated measures was conducted with Attention (DA, FA), Material (random shapes, words) and TMS Condition (right DLPFC, left DLPFC, sham) as within subject-factors. Post-hoc analyses were conducted using Fisher's LSD test.

## 3. Results

Performance obtained in recognition following each encoding condition is illustrated in Table 1.

### 3.1. Hits

A significant Attention x TMS Condition interaction was found [F(2,30) = 3.46, $P = 0.04$]. The interaction is illustrated in Fig. 2. Post-hoc analyses detected that participants recalled significantly fewer items after TMS over the left DLPFC than over the right DLPFC during encoding under FA ($P = 0.03$), but not versus the sham FA encoding condition. In contrast, participants recalled fewer items after TMS over the right DLPFC during encoding under DA as compared to the sham DA encoding condition ($P = 0.05$), but not versus left TMS. The main effect of Attention was significant [F(1,15) = 56.76, $P < 0.001$].

The Material x TMS Condition interaction did not reach the level of significance [F(1, 30) = 3.46, $P =$ 0.14]. Finally, ANOVA failed to detect any main effect of TMS Condition or Material [F(2,30) = 1.98, $P =$ 0.15, and F(1, 30) = 2.95, $P = 0.11$, respectively] and any interaction between Attention, TMS Condition and Material (F < 1).

### 3.2. Discrimination rates

ANOVA applied on discrimination rates detected a main effect of Attention [F(1,15) = 24.05, $P < 0.001$], indicating that the discrimination rate was higher after encoding under FA (63.97 % ± 18.7) than encoding under DA (50.7 % ± 20.16). A main effect of Material was also significant [F(1,15) = 30.92, $P < 0.001$], reflecting a higher discrimination rate for words (68.86 % ± 17.43) than for random shapes (45.81 % ± 23.05).

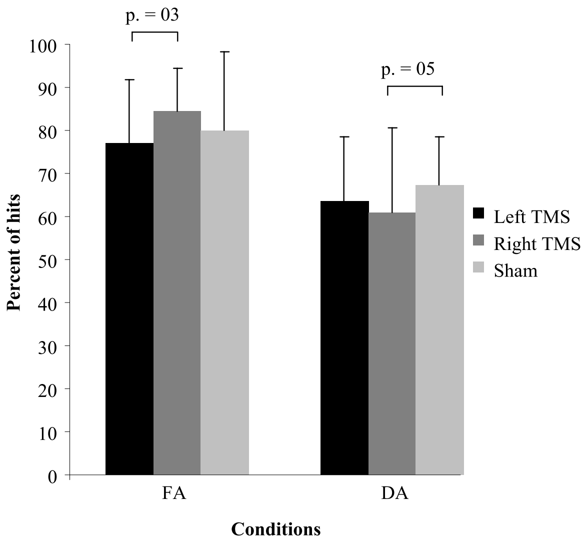

Fig. 2. Means (and standard deviations) of hit percent after encoding under full attention (FA) or divided attention (DA) according to TMS condition. Data are collapsed for both verbal and non-verbal material under DA and FA conditions. Participants recalled fewer items after TMS over the left DLPFC as compared to TMS over the right DLPFC during FA encoding ($P = 0.03$), but not compared to the sham encoding condition. In contrast, when TMS was applied during encoding under DA over the right DLPFC, participants recalled subsequently fewer items as compared to the sham encoding condition ($P$= 0.05), but not as compared to TMS over the left DLPFC. These effects are seen for every type of material.

Furthermore, there was no Attention x TMS Condition interaction [F(2,30) = 3.07, $P = 0.07$] and no TMS Condition main effect [F(2,30) = 1.12, $P = 0.34$]. No other interaction was significant (F < 1).

### 3.3. False alarms

The Material main effect was significant [F(1,15) = 27.11, $P < 0.001$], indicating that more false alarms were produced for random shapes (22.63% ± 14.11) than for words (5.82 % ± 4.66). There was no main effect of Attention [F(1, 15) = 1.24, $P = 0.28$]. No other interaction or effects were significant (F < 1).

### 3.4. Response time of hits

The interaction Attention, Material by TMS Condition was significant [F(1,30) = 3.74, $P = 0.04$]. Post-hoc analyses revealed that for random shapes encoded under FA, response time was longer after TMS over the left DLPFC than in the sham condition ($P = 0.04$). After encoding under DA, the response time for random shapes was longer after TMS over the right DLPFC than after TMS over the left DLPFC ($P < 0.005$). It

Table 1
Means and standard deviations obtained at the recognition paradigm according to the TMS encoding conditions

| | | Full attention | | | | | | Divided attention | | | | | |
|---|---|---|---|---|---|---|---|---|---|---|---|---|---|
| | | Non-verbal | | | Verbal | | | Non-verbal | | | Verbal | | |
| Indices | | Left DLPFC | Right DLPFC | Sham | Left DLPFC | Right DLPFC | Sham | Left DLPFC | Right DLPFC | Sham | Left DLPFC | Right DLPFC | Sham |
| Hits (%) | Mean | 75.97 | 79.38 | 78.13 | 78.13 | 89.22 | 81.88 | 61.88 | 55.00 | 65.63 | 65.28 | 67.01 | 69.10 |
| | StDev | 18.14 | 14.36 | 18.70 | 15.59 | 11.24 | 15.15 | 19.74 | 23.66 | 19.31 | 21.11 | 20.77 | 22.38 |
| FA (%) | Mean | 26.32 | 23.13 | 23.13 | 8.39 | 6.25 | 5.14 | 18.75 | 23.13 | 21.39 | 4.44 | 4.38 | 6.32 |
| | StDev | 19.22 | 19.91 | 15.80 | 9.23 | 8.06 | 12.71 | 17.46 | 21.52 | 19.96 | 6.36 | 6.29 | 12.61 |
| DR (%) | Mean | 49.65 | 56.25 | 55.00 | 69.73 | 82.97 | 76.74 | 43.13 | 31.88 | 44.24 | 60.83 | 62.64 | 62.78 |
| | StDev | 29.74 | 24.46 | 25.82 | 19.55 | 17.92 | 24.16 | 22.13 | 31.46 | 27.91 | 23.00 | 24.86 | 22.78 |
| Hits RT | Mean | 908.95 | 902.17 | 846.56 | 815.39 | 777.65 | 830.39 | 843.63 | 941.40 | 905.72 | 827.13 | 834.47 | 808.69 |
| (ms) | StDev | 187.54 | 138.32 | 175.98 | 149.20 | 127.45 | 122.32 | 123.01 | 139.74 | 143.32 | 146.06 | 99.80 | 140.35 |

DLPFC: Dorsolateral Prefrontal Cortex; FA: False Alarms; DR: Discrimination Rate; RT: Response Times.

was also longer in the sham DA encoding condition than after left DLPFC TMS ($P = 0.03$). There was also a Material main effect [F(1,15) = 7,73, $P = 0.01$]. The Attention x TMS Condition interaction was not significant [F(1,30) = 2.47, $P = 0.11$], neither the Material x Condition interaction [F(1, 30) = 2.54, $P = 0.11$]. No other interactions or effects were detected (F $<$ 1).

### 3.5. *Digit monitoring task performance*

An ANOVA Material (random shapes, words) by TMS Condition (left DLPFC, right DLPFC, sham) revealed only a Material main effect [F(1,30) = 6.15, $P = 0.03$], indicating fewer responses at the digit monitoring task when random shapes (93.33% $\pm$ 8.15) were presented as compared to words (96.73% $\pm$ 4.55). Neither Condition effect nor interaction was significant (F $<$ 1).

## 4. Discussion

The present study aimed to investigate the involvement of the DLPFC in the attentional resources during encoding according to the type of material (verbal or non-verbal). For this purpose, safe focal paired-pulse TMS was applied to interfere transiently with either the left or right DLPFC during encoding under FA or DA of words or random shapes. Our results detected a dichotomy for the TMS interference of DLPFC during encoding. Indeed, under FA, TMS over the left DLPFC interfered detrimentally on memory performance whereas TMS over the right DLPFC interfered only under DA, regardless of the type of material. The discussion that follows presents the concurrent involvement of the left and right DLPFC in encoding under FA and DA, respectively.

We found that TMS over the left DLPFC during encoding under FA decreased hit percent when compared to TMS over the right DLPFC, for both random shapes and words. Hit response time was also longer after TMS over left DLPFC than in the sham condition. These findings are in agreement with previous neuroimaging data showing that the left DLPFC is preferentially activated in encoding in healthy young individuals [2,22]. Impaired recall performance has also been reported after rTMS over the left DLPFC during encoding compared to rTMS over the right DLPFC for verbalizable material such as a short story [35], unrelated words [20,41] or indoor/outdoor pictures [36]. Our results extend further that the left DLPFC is not only activated during encoding under FA, but it is also necessary for successful encoding of both unrelated verbal and non-verbal material. Surprisingly, the inhibitory effects of TMS over the left DLPFC during FA encoding were detected only for the hits, but not for the discrimination rates. Previous neuroimaging studies showed that DLPFC activations in organizational or elaborative strategies during encoding are a predictive value of subsequent long-term memory performance [9, 31] (for a review, see [10]). In our study, the inhibitory TMS effect over the left DLPFC during FA encoding would thus exert a detrimental effect on strategies that contribute to successful encoding. Even if our instructions did not explicitly orient participants to adopt mnemonic strategies, our participants may have applied them. This is in agreement with impaired performance on different memory indices in patients with left posterior DLPFC lesions (BA 44, 9, 46) [1]. According to these authors, these deficits are related to difficulties in applying strategies of encoding. Findings from complementary approaches therefore demonstrated that the left DLPFC plays an important role in episodic encoding.

During encoding under DA, hit percent was lower after TMS over the right DLPFC than after TMS over the left DLPFC. Response time for non-verbal material was also longer after TMS over the right DLPFC than after TMS over the left DLPFC. Contrary to encoding under FA, we found that the inhibitory effects of TMS on memory performance when delivered over the left DLPFC are less pronounced for encoding under DA. In this way, neuroimaging studies reported that the left DLPFC activations were decreased under DA for an encoding task that required explicit semantic organizational or associative strategies of related words [17,22]. Conversely, our results brought about the evidence of the role of the right DLPFC during encoding under DA. This may be related to the involvement of control executive system in both the encoding of unrelated items that would greater solicit self-initiated elaborative strategy than related items, as well as the secondary task. This right lateralized DLPFC contribution under DA may reflect item-related processes during encoding as already evidenced by means of techniques investigating item-related activity (e.g., event-related potentials, event-related functional magnetic resonance imagery) rather than task-related activity (e.g., PET). Indeed, using event-related potentials, Mangels, Picton and Craik [28] reported greater right frontal sustained activity for recalled and remembered words when encoded concomitantly to a difficult secondary task. This experimental condition depended on efficient self-initiative strategy and involved an executive control that was necessary for the active maintenance and manipulation of information. Using event-related functional magnetic resonance imaging, Uncapher and Rugg [47] even found a trade-off on the right DLPFC activations indicated by a reduced study-item activity contrasting with an increased auditory-item activity in the difficult, relative to easy, secondary task condition. In the present study, TMS over the right DLPFC may have therefore interfered with the right DLPFC item-activity involved during both the encoding and secondary tasks, independently of the type of the material to encode.

Some methodological considerations specific to our study, such as the frequency TMS and type of TMS coil used need to be addressed. Surprisingly, in our study, subsequent recall memory performance decreased only when TMS was applied over left DLPFC during FA encoding compared to TMS over right DLPFC. In contrast, this difference did not reach the significant level when TMS was applied over left DLPFC compared to the sham condition. The HERA model postulates that the left PFC is preferentially more involved in encoding of episodic memory than the right PFC [46]. However, the Nyberg et al.'s meta-analysis of neuroimaging studies supported that the predominant activation of the left PFC for encoding was detected in comparison to reference tasks [31]. Using rTMS, in agreement with our findings, Rami et al. [35] also found this asymmetry between left and right DLPFC, but not with sham or baseline conditions. One explanation for the discrepancy between these TMS and neuroimaging studies could be the disruption of the balance of the interhemispheric activity by interferential TMS. Actually, it is known that various cortical functions depend on a balance between both hemispheres, controlled by reciprocal interhemispheric inhibition [19,27]. Monohemispheric application of low-frequency rTMS that decreases the cortical excitability (i.e., inhibition) can disrupt this balance and disinhibit the contralateral non-stimulated hemisphere via transcallosal pathways [33]. Conversely, high-frequency rTMS that increases locally the excitability disrupts the balance by exacerbating the inhibition over the contralateral non-stimulated hemisphere. In the memory field, the increase of excitability in the left DLPFC during encoding by high-frequency rTMS (e.g., 20 Hz) may have thus inhibited the right DLPFC, leading to a worsening of subsequent recall memory performance [18,20,36,37,41,43]. In contrast, low-frequency rTMS (1 Hz) of the right posterior ventrolateral PFC during encoding led to a better memory performance as compared to left TMS [24]: transcallosal disinhibition of the left PFC may be the physiological substrate of enhanced encoding, in line with the authors' suggestion of a transient 'disengagement' of right PFC. In our protocol, we used inhibitory low-frequency paired-pulse TMS (0.5 Hz, 3-ms interstimulus interval) over either the left or the right DLPFC during encoding under FA. Right TMS (i.e., decrease of right DLPFC excitability) may have disinhibited the left DLPFC (via the decrease of transcallosal inhibition) thus leading to improved encoding compared to the sham condition. This may explain why the left TMS during encoding under FA worsened subsequent recall memory performance only when compared to the right TMS, but not to the sham condition. Therefore, it cannot be ruled out that low-frequency TMS induces weaker memory performance decrease than high-frequency TMS. Following this reasoning, Rami et al. [35] reported lower subsequent memory performance after TMS over the left DLPFC, at a rate of 1-Hz (low-frequency) as compared to 5 Hz (high-frequency). In contrast, in our study, memory performance was dropped by right

TMS during encoding under DA (thus supporting the engagement of the right DLPFC in encoding under DA) but only when compared to the sham condition and not to the left TMS. TMS over the left DLPFC (i.e., disinhibiting further the right DLPFC already engaged in encoding) may have altered performance likely by a disruption of the activity balance between both DLPFC. This may therefore support a cooperative involvement of both sides in encoding under DA.

One another methodological issue concerns the type of coil used. The TMS double cone coil we employed usually enables recruitment of deep structures [40]. In this way, our TMS protocol may have influenced areas below the target DLPFC between 3 to 4 cm in depth. This is quite different from most memory studies having used a flat figure-of-eight coil to induce inhibitory effects in specific areas [16,18,20,36]. Indeed, the electric current under the flat coil is circumscribed to superficial (cortical) brain areas and is thus vertically more focal than the double cone coil [13]. Conversely, the flat coil is less focal horizontally (i.e., recruitment of adjacent areas under the coil wings) than the double cone coil whose wings curvature enables a stronger current in the middle point of the coil [21]. However, we can argue that the subthreshold paired-pulse TMS paradigm we used to induce DLPFC inhibition produced the largest effects at the site of stimulation with fewer effects from the stimulated site to distant areas than rTMS did [30]. Indeed, when coupling both paired-pulse TMS and fMRI recordings, Bestman et al. [4] have shown that paired-pulse TMS did not activate distant areas when delivered at a subthreshold intensity. Since we used a subthreshold 3-ms paired-pulse TMS paradigm, we are confident that TMS direct effects were circumscribed to DLPFC area. Nevertheless, it can be argued that indirect influence on remote areas may have occurred at the functional connections that DLPFC shares with, for example, the cingulate cortex and thalamic nuclei [29]. In our study, because TMS effects in FA encoding varied according to the laterality of the stimulations in agreement with the HERA model, our findings show influence in favour of circumscribed inhibitory effects induced by paired-pulse TMS over DLPFC. In the future, studies combining neuroimaging techniques and paired-pulse TMS may be relevant to probe how paired-pulse TMS affects neural mechanisms of cognitive functions.

In conclusion, our interferential TMS study confirms that the left DLPFC plays an important role in FA encoding. Originally, our results provide the first new evidence of the critical concurrent involvement of the right DLPFC in successful encoding with a secondary task for both unrelated verbal and non-verbal materials. Interestingly, the right DLPFC may benefit from neural/cognitive compensatory recovery after an acquired brain injury since the memory performance of patients with anterior injuries is not impaired after encoding under DA [3,48]. However, this needs to be confirmed because in these studies patients were not classified according to the specific PFC subareas that were altered. Future studies in patients with acquired brain injury should thus focus on cerebral plasticity by coupling neuropsychological investigations with neuroimaging or event-related potential techniques.

## Acknowledgments

This study was supported by a National Science and Engineering Research Council (299425-04) grant, a research scholarship from Fonds de la Recherche en Santé du Québec as well as a grant for summer students from the Quebec Rehabilitation Research Network (REPAR) to Sophie Blanchet. The authors thank Gabrielle Chabot and Caroline Gagnon for their assistance in data collection.
